# The association between air pollution and the severity at diagnosis and progression of systemic sclerosis-associated interstitial lung disease: results from the retrospective ScleroPol study

**DOI:** 10.1186/s12931-023-02463-w

**Published:** 2023-06-08

**Authors:** Anaïs Roeser, Lucile Sese, Guillaume Chassagnon, Benjamin Chaigne, Bertrand Dunogue, Stéphane Tran Ba, Salma Jebri, Pierre-Yves Brillet, Marie Pierre Revel, Frédérique Aubourg, Robin Dhote, Frédéric Caux, Isabella Annesi-Maesano, Luc Mouthon, Hilario Nunes, Yurdagül Uzunhan

**Affiliations:** 1 Department of Pulmonology, Assistance Publique-Hôpitaux de Paris (APHP), Avicenne Hospital, Bobigny, France; 2 Department of Radiology A, Assistance Publique-Hôpitaux de Paris (APHP), Cochin Hospital, Paris, France; 3 Department of Internal Medicine, Assistance Publique-Hôpitaux de Paris (APHP), Cochin Hospital, Paris, France; 4 Department of Radiology, Assistance Publique-Hôpitaux de Paris (APHP), Avicenne Hospital, Bobigny, France; 5 Department of Physiology, Assistance Publique-Hôpitaux de Paris (APHP), Cochin Hospital, Paris, France; 6 Department of Internal Medicine, Assistance Publique-Hôpitaux de Paris (APHP), Avicenne Hospital, Paris, France; 7 Department of Dermatology, Assistance Publique-Hôpitaux de Paris (APHP), Avicenne Hospital, Paris, France; 8 INSERM, Department of Allergic and Respiratory Disease, Montpellier University Hospital, Institute Desbrest of Epidemiology and Public Health, University of Montpellier, Montpellier, France; 9 INSERM UMR1272 Hypoxie et poumon, Paris 13 - Université Paris Nord, Bobigny, France

**Keywords:** Systemic sclerosis, Scleroderma, Interstitial lung disease, Air pollution, Ozone, Particulate matter, Nitrogen dioxide, Severity, Progression, CHIMERE

## Abstract

**Objective:**

To investigate the association of air pollution exposure with the severity of interstitial lung disease (ILD) at diagnosis and ILD progression among patients with systemic sclerosis (SSc)-associated ILD.

**Methods:**

We conducted a retrospective two-center study of patients with SSc-associated ILD diagnosed between 2006 and 2019. Exposure to the air pollutants particulate matter of up to 10 and 2.5 µm in diameter (PM_10_, PM_2.5_), nitrogen dioxide (NO_2_), and ozone (O_3_) was assessed at the geolocalization coordinates of the patients’ residential address. Logistic regression models were used to evaluate the association between air pollution and severity at diagnosis according to the Goh staging algorithm, and progression at 12 and 24 months.

**Results:**

We included 181 patients, 80% of whom were women; 44% had diffuse cutaneous scleroderma, and 56% had anti-topoisomerase I antibodies. ILD was extensive, according to the Goh staging algorithm, in 29% of patients. O_3_ exposure was associated with the presence of extensive ILD at diagnosis (adjusted OR: 1.12, 95% CI 1.05–1.21; *p* value = 0.002). At 12 and 24 months, progression was noted in 27/105 (26%) and 48/113 (43%) patients, respectively. O_3_ exposure was associated with progression at 24 months (adjusted OR: 1.10, 95% CI 1.02–1.19; *p* value = 0.02). We found no association between exposure to other air pollutants and severity at diagnosis and progression.

**Conclusion:**

Our findings suggest that high levels of O_3_ exposure are associated with more severe SSc-associated ILD at diagnosis, and progression at 24 months.

**Supplementary Information:**

The online version contains supplementary material available at 10.1186/s12931-023-02463-w.

## Introduction

Systemic sclerosis (SSc) is a systemic disease characterized by autoimmune features, and endothelial and fibroblast dysfunctions, resulting in vasculopathy and tissue fibrosis. Interstitial lung disease (ILD) is common in SSc. In a recent nationwide cohort study in Norway, high-resolution computed tomography (HRCT) showed that half the patients had ILD [1]. ILD has a major impact on the morbidity and mortality of SSc patients, one third of whom die from pulmonary fibrosis [2]. The course of SSc-associated ILD is heterogeneous [3]. Extensive lung parenchyma involvement according to the Goh staging algorithm [4] and short-term functional decline [5, 6] have been described as predictors of poorer survival, but the factors underlying the prognostic heterogeneity between patients are not fully understood. Nevertheless, ethnic, immunological and phenotypic characteristics of SSc, such as Afro-Caribbean origin, anti-topoisomerase I antibodies, esophageal diameter, reflux/dysphagia symptoms, modified Rodnan skin score, diffuse cutaneous phenotype (dcSSc) and being male [3, 7–9], have been shown to be associated with ILD severity and progression.

Air pollution has been implicated in idiopathic pulmonary fibrosis (IPF) in a number of studies. The incidence of IPF was associated with levels of exposure to nitrogen dioxide (NO_2_) and particulate matter of up to 2.5 µm in diameter (PM_2.5_) [10, 11]. Disease severity has been linked to exposure to particulate matter of up to 10 µm or up to 2.5 µm in diameter (PM_10_ and PM_2.5_) [12]. Disease exacerbations were linked to exposure to ozone (O_3_), NO_2_, PM_10_, and PM_2.5_ [13–16], and functional decline with exposure to PM_10_ [14]_._ Mortality was associated with exposure to PM_10_, PM_2.5_ and NO_2_ [12, 14, 17–19]. A role for air pollution in SSc was first suggested by a British study reporting a higher prevalence of SSc in the London region, particularly in boroughs close to airports, than in the West Midlands [20]. More recently, an Italian study on 88 SSc patients found that benzene exposure was positively correlated with skin score and inversely correlated with the diffusion of carbon monoxide in the lung (DLCO) [21]. However, to our knowledge, no larger-scale study has evaluated the impact of air pollution on SSc-associated ILD.

We conducted a retrospective study to evaluate the contribution of the principal air pollutants (PM_10_, PM_2.5_, NO_2_, and O_3_) to the natural course of SSc-associated ILD. The primary objective was to determine the association between air pollution exposure and disease severity at diagnosis according to the Goh staging algorithm [4]. The secondary objective was to evaluate the impact of air pollution on disease progression.

## Patients and methods

### Patients

The study population consisted of patients identified from the French hospital discharge database (*Programme de Médicalisation des Systèmes d'Information* [PMSI]) seen from January 1 2006 to December 31 2019 at two French centers in the Paris area (the internal medicine department of Cochin Hospital, Paris, and the respiratory medicine, internal medicine and dermatology departments of Avicenne Hospital, Bobigny). For inclusion in the study, patients had to have SSc, defined according to the 2013 American College of Rheumatology/European League Against Rheumatism (ACR/EULAR) classification criteria [22] and ILD, diagnosed on HRCT, with PFT results available from the 3 months immediately before or after SSc-associated ILD diagnosis. The patients also had to be at least 18 years old at the time of SSc-associated ILD diagnosis. Patients living abroad or in French overseas territories were excluded.

Scleroderma phenotype (dcSSc, limited cutaneous SSc, *sine scleroderma* SSc), associated non-pulmonary organ’s involvements, specific auto-antibodies, time between ILD diagnosis and first non-Raynaud symptom were collected. Follow-up PFT results were collected at 6, 12, 18 and 24 (± 3) months and last follow-up. Pulmonary volumes and flow (total lung capacity [TLC], forced vital capacity [FVC], and forced expiratory volume in 1 s [FEV1]) were calculated as a percentage of predicted values with the Global Lung Initiative equations. A recourse to lung transplantation and the occurrence of death were also recorded. Initial HRCT data and HRCT data obtained at 2 years of follow-up (HRCT performed on the date closest to 2 years) were reviewed and a consensus interpretation was reached between expert radiologists blinded to clinical symptoms, autoantibody subtype and treatment. The expert radiologists concerned had 7 (S.T.B.), 7 (G.C.), 10 (S.J.), and 20 (P-Y.B.) years of experience in chest imaging. The extent of lesions (honeycombing, reticulations, ground-glass opacities and/or consolidations) was quantified over the whole lung, with the method described by Akira et al. [23], in which the lungs are divided into six zones, three for each lung (upper zone: above the carina, middle zone: between the carina and the inferior pulmonary veins, lower zone: below the inferior pulmonary veins). The overall extent of parenchymal abnormalities is estimated by averaging the estimated extent of the disease in the six zones. The radiologists attributed an ILD pattern from the following list to each HRCT image: usual interstitial pneumonia (UIP), organizing pneumonia (OP), non-specific interstitial pneumonia (NSIP), early ILD, unclassifiable ILD. Patterns classified as UIP or probable UIP according to American Thoracic Society, European Respiratory Society, Japanese Respiratory Society, and Asociación Latinoamericana de Tórax (ATS/ERS/JRS/ALAT) Clinical Practice Guidelines were classified here as UIP [24]. A NSIP pattern was attributed to HRCT with predominant ground-glass opacities, with or without reticulations or traction bronchiectasis, with a predominantly basal distribution, and with no more than minimal honeycombing [25]. An OP pattern was defined as patchy, often migratory consolidation in a subpleural, peribronchial, or band-like pattern, commonly associated with ground-glass opacity according to the ATS/ERS criteria [26]. Early and unclassifiable ILD were labelled as indeterminate ILD.

### Exposure to air pollution

The concentrations of air pollutants were obtained with the CHIMERE chemistry-transport model for mainland France [27]. This model uses meteorological fields, primary pollutant emissions, and chemical boundary conditions to calculate the atmospheric concentrations of gas and particles over local to continental domains (with a resolution from 1 km to 1 degree). In this study, we used this model to obtain, for the geolocalized address of each patient, the mean annual concentrations of NO_2_, O_3_, and PM_10_ available from 2000 to 2019 and of PM_2.5_ available from 2009 to 2019, with a resolution of 2 km. Two periods of exposure were defined: (1) exposure before ILD diagnosis was calculated by determining the mean concentrations of NO_2_, O_3_, PM_10_ and PM_2.5_ for the 5 years preceding SSc-associated ILD diagnosis; (2) exposure after ILD diagnosis was estimated from the mean annual concentrations in the year of ILD diagnosis.

### Statistical analysis

Data were expressed as absolute numbers (percentages) for categorical variables and as the median (interquartile range, IQR) or mean (standard deviation, SD) for quantitative variables.

#### Severity at diagnosis

Multiple logistic regression models were constructed to evaluate the impact of pre-ILD diagnosis air pollution on ILD severity at diagnosis, as evaluated with the Goh staging algorithm [4]. ILD was classified as extensive or limited: cases with an ILD extension on HRCT > 30% were considered extensive; cases with an extension ≤ 10% were considered limited; patients for whom extension was intermediate were classified as having extensive ILD if FVC < 70%, and limited ILD if FVC ≥ 70%.

We performed a sensitivity analysis to evaluate the impact of a change in judgement criteria, using other severity parameters: TLC < 70%, FVC < 70%, DLCO < 40%, composite physiological index (CPI) > 40 and ILD extension on HRCT > 10% in logistic regression models, and considering TLC, FVC, DLCO, CPI and ILD extent on HRCT at baseline as continuous variables in linear mixed models.

#### Evolution of ILD

We used two methods to determine whether air pollution exposure after ILD diagnosis was associated with evolution of ILD during follow-up.

First, we used multiple logistic regression models and Cox proportional hazard models to evaluate the association between air pollution exposure and the occurrence of progression within 24 months following ILD diagnosis. Functional decline was calculated based on relative changes in FVC or DLCO [(baseline value − follow-up value)/baseline value], with FVC measured in liters and DLCO as a percentage of the predicted value. Progression was defined as a relative decrease of at least 10% in FVC compared to baseline; or as a relative decrease between 5 and 10% in FVC plus a relative decrease in DLCO of at least 15% according to Outcome Measures in Rheumatology (OMERACT) Connective Tissue Disease—ILD criteria [28]. Patients died or who had required lung transplantation during the considered follow-up period (12 or 24 months) were considered to have undergone progression.

A sensitivity analysis was performed with functional and radiological surrogates, defined as relative decline in FVC ≥ 10% or ≥ 5% and relative decline in DLCO ≥ 15%, ≥ 10% or ≥ 7.5% at 24 months, and the occurrence of radiological progression at 24 months. Radiological progression was defined in accordance with the 2022 ATS/ERS/JRS/ALAT Guidelines [29].

Second, we used linear mixed models to identify predictors of change in FVC (absolute decline in mL) or DLCO (absolute decline in % of predicted) over time. This model included a single, subject-level random effect, and fixed effects for potential predictors of change and time.

A Cox proportional hazards model was used to estimate the impact of air pollution on transplant-free survival, considering the time from ILD diagnosis to death or lung transplantation or last follow-up.

All multiple logistic regression models, linear mixed models and Cox proportional hazards models were adjusted for factors identified in univariate analysis, which were included in the multivariate model if the *p* value was < 0.2 (|t| value > 1.3 for mixed linear models). Final multivariate models included factors associated with the outcome in multivariate analysis (*p* value < 0.2 or |t| value > 1.3 for mixed linear models). Potential predictors analyzed were factors already shown to be associated with SSc-associated ILD severity, or progression of fibrosing ILD [30], or potential confounders: sex, age at ILD diagnosis, tobacco smoking, dcSSc, positive anti-topoisomerase I antibodies, UIP pattern, time from first non-Raynaud phenomenon, and for follow-up outcomes: baseline FVC and DLCO, ILD extension on initial HRCT, and initiation of an immunosuppressive therapy. We also considered patient’s continent of birth, socio-economic status, evaluated through the socio-professional category defined by the French *Institut national de la statistique et des études économiques* (INSEE), and year of ILD diagnosis as potential confounders for health outcomes.

Statistical analysis was performed with R software V.4.1.2, and statistical significance was defined as a *p* value < 0.05 (|*t*| value > 2 for mixed linear models).

### Ethical considerations

This study received Institutional Review Board approval (Comité Local d’Ethique pour la Recherche Clinique des HUPSSD, CLEA-2020-150) and the requirement for signed informed consent was waived according to French legislation (CNIL Reference methodology).

## Results

### Study population

We screened 269 patients with SSc-associated ILD defined on HRCT. We excluded 88 cases because they had ILD diagnosed before 2006 (*n* = 39), had no PFT data from a period within three months of diagnosis (*n* = 36), were less than 18 years old (*n* = 1), or were living abroad or in French overseas territories (*n* = 12) (Additional file 1: Fig. S1). We included 181 patients, 79.6% of whom were female. Most of patients were born in Europe (58%), 30.9% were born in Africa, and 6.6% in Asia. Thirteen percent of patients belonged to the working-class, and 6% had no professional activity. Median age at SSc diagnosis was of 53 years (IQR: 42.5–64 years); 44.2% had dcSSc, with anti-topoisomerase I antibodies in 55.8% and anti-centromere antibodies in 11.0% (Table 1). ILD was extensive in 53 of 181 cases (29.3%). Median (IQR) FVC at diagnosis was 78.5% (63.7–93.8), and median (IQR) DLCO was 55% (42–66%). NSIP was the most frequent radiological pattern (63.5%).

**Table 1:** Characteristics of the patients with systemic sclerosis-associated interstitial lung disease included in the study

	Total population *N* = 181	Extensive ILD *N* = 53	Limited ILD *N* = 128
Female, *n* (%)	144 (79.6)	40 (75.5)	104 (81.3)
Smoking status			
Never smoker, *n* (%)	113/172 (65.7)	38/51 (74.5)	75/121 (62.0)
Former smoker, *n* (%)	16/172 (9.3)	2/51 (3.9)	14/121 (11.6)
Current smoker, *n* (%)	43/172 (25.0)	11/51 (21.6)	32/121 (26.4)
Age at SSc diagnosis (years), median (IQR)	53 (42.5–64)	54 (44.5–64)	53 (42–64.8)
Cutaneous phenotype			
Diffuse cutaneous, *n* (%)	80 (44.2)	26 (49.1)	54 (42.2)
Limited cutaneous, *n* (%)	89 (46.4)	25 (47.2)	64 (50.0)
Sine scleroderma, *n* (%)	12 (6.6)	2 (3.8)	10 (7.8)
ScS involvement			
Gastrointestinal, *n* (%)	139 (76.8)	42 (79.2)	97 (75.8)
Cardiac, *n* (%)	19 (10.5)	7 (13.2)	12 (9.4)
Muscular, *n* (%)	14 (7.7)	3 (5.7)	11 (8.6)
Renal, *n* (%)	5 (2.8)	1 (1.9)	4 (3.1)
Autoantibodies			
Anti-centromere, *n* (%)	20 (11.0)	1 (1.9)	19 (14.8)
Anti-topoisomerase I, *n* (%)	101 (55.8)	37 (69.8)	64 (50.0)
Anti-RNA polymerase III, *n* (%)	11 (6.1)	4 (7.5)	7 (5.5)
Time from first non-Raynaud symptom (years) to ILD diagnosis, median (IQR)	2 (1–5)	2 (1–4)	2 (1–7)
Pulmonary function			
FVC (% predicted), median (IQR)	78.5 (63.7–93.8)	61.7 (50.5–69.8)	84.1 (74.5–98.6)
TLC (% predicted), median (IQR)	83.4 (71.8–99.2)	70.6 (61.9–76.0)	92.4 (78.5–102.2)
FEV1 (% predicted), median (IQR)	81.1 (67.5–94.9)	67.9 (57.4–74.3)	86.1 (73.0–97.6)
DLCO (% predicted), median (IQR)	55.0 (42.0–66.0)	43.5 (33.8–51.0)	61 (49.0–71.5)
Composite physiological index, median (IQR)	40.9 (28.8–50.0)	52.0 (47.1–59.5)	35.4 (25.8–45.1)
Radiological pattern			
UIP	13 (7.2)	6 (12.1)	7 (5.5)
NSIP	115 (63.5)	43 (75.9)	72 (56.2)
Indeterminate ILD	53 (29.3)	4 (10.3)	49 (38.3)
Extent of ILD (%), median (IQR)	10 (5–20)	30 (16–35)	5 (5–12)
Emphysema association, *n* (%)	34 (18.8)	9 (17.0)	25 (19.5)
Extent of emphysema (%), median (IQR)	5 (3–10)	3 (3–7.5)	5 (3–10)
Hiatal hernia	29 (16.0)	9 (17.2)	20 (15.6)
Esophageal dilation	92 (47.9)	30 (51.7)	62 (48.4)
Immunosuppressive therapy initiation^a^, n (%)	81 (44.8)	31 (58.5)	50 (39.1)

*DLCO* diffusion capacity for carbon monoxide across the lung, *FEV1* forced expiratory volume in one second, *FVC* forced vital capacity, *ILD* interstitial lung disease, *NSIP* nonspecific interstitial pneumonia, *TLC* total lung capacity, *UIP* usual interstitial pneumonia

^a^Initiation of any immunosuppressive therapy after ILD diagnosis (excluding steroids)

**Fig. 1:**
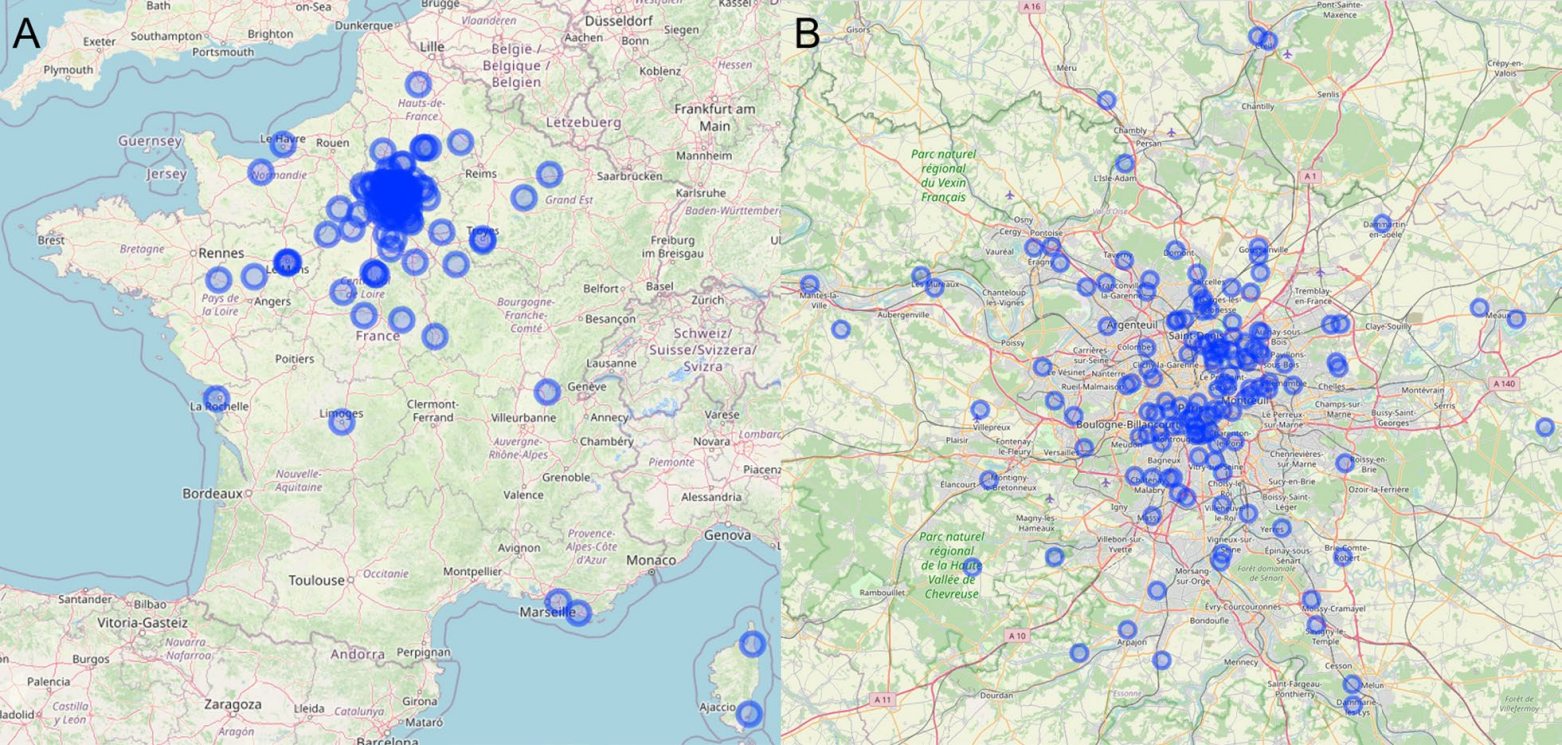
Distribution of the patients included in the study across mainland France. **A** Global distribution of the patients’ residential addresses in mainland France; **B** focus on the Parisian region (Ile-de-France)

All the patients included in this study were resident in mainland France, and 139/181 (77%) were living in the Paris region (Fig. 1). The mean (SD) exposure levels during the 5 years preceding ILD diagnosis for the study population were 27.9 (8.4) µg/m^3^ for NO_2_ (range 9.9–42.2), 44.2 (6.0) µg/m^3^ for O_3_ (range 36.5–74.6), 23.5 (3.3) µg/m^3^ for PM_10_ (range 15.6–30.0) and 15.6 (2.4) µg/m^3^ for PM_2.5_ (range 9.6–20.3). About 99.5% of patients had pre-ILD diagnosis exposure levels above the recent WHO recommendations for NO_2_, and 100% had pre-ILD diagnosis exposure levels above WHO recommendations for PM_10_ and PM_2.5_ [31].

### ILD severity at diagnosis and air pollution exposure

**Fig. 2:**
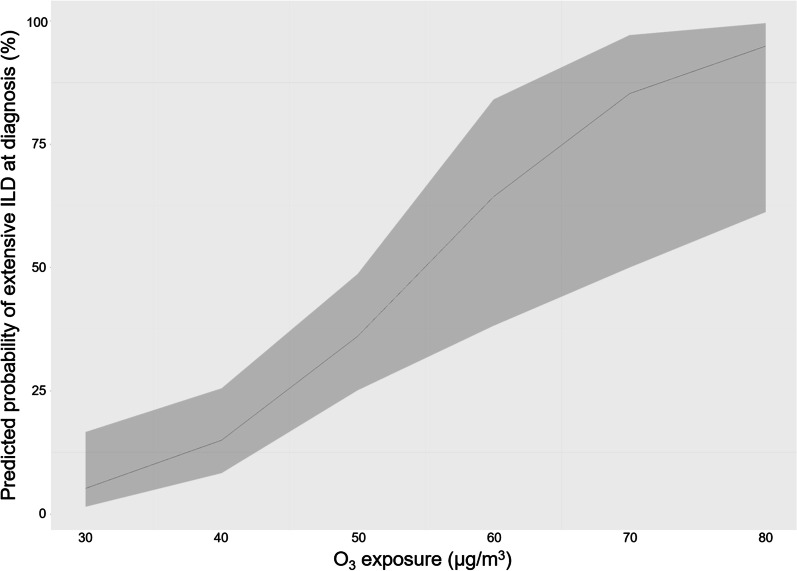
Predicted probability of presence of an extensive interstitial lung disease (ILD) at diagnosis according to ozone exposure in final multivariate logistic regression model

In univariate analysis, pre-ILD diagnosis O_3_ exposure tended to be associated with the presence of extensive ILD at diagnosis (OR: 1.05, 95% CI 1.00–1.11; *p* value = 0.06) (Additional file 1: Table S1). Parameters included in the final multivariate model were those associated with the presence of an extensive ILD in multivariate analysis: a non-European place of birth (adjusted OR for birth in Europe: 0.23, 95% CI 0.11–0.63; *p* value = 0.003), tobacco smoking (adjusted OR: 0.33, 95% CI 0.10–0.92; *p* value = 0.04), anti-topoisomerase I antibodies (adjusted OR: 4.56, 95% CI 1.75–13.43; *p* value = 0.003), UIP pattern (adjusted OR: 3.48, 95% CI 0.64–18.45; *p* value = 0.14), time between first non-Raynaud symptom and ILD diagnosis (adjusted OR: 0.93, 95% CI 0.84–1.01; *p* value = 0.16) and year of ILD diagnosis (adjusted OR: 1.08, 95% CI 0.97–1.22; *p* value = 0.19). In this final multivariate model, O_3_ exposure was significantly associated with the presence of extensive ILD (adjusted OR: 1.12, 95% CI 1.05–1.21; *p* value = 0.002) (Table 2). Thus, the predicted probability of extensive ILD for mean exposures of 30 µg/m^3^ and 60 µg/m^3^ were respectively 5 (95% CI 1–17) % and 64 (95% CI 38–84) % (Fig. 2).

**Table 2:** Association of air pollution (pre-diagnosis exposure) with the severity of SSc-associated ILD at diagnosis (extensive ILD)

	OR	*p* value
NO_2_	0.95 (0.91–1.00)	0.08
O_3_	1.12 (1.05–1.21)	0.002
PM_10_	0.91 (0.75–1.11)	0.36
PM_2.5_	0.87 (0.64–1.20)	0.39

Logistic regression models adjusted for birth in Europe, tobacco smoking, anti-topoisomerase I antibodies positivity, usual interstitial pneumonia pattern, time between first non-Raynaud symptom and ILD diagnosis, and year of ILD diagnosis

ILD: interstitial lung disease; NO_2_: nitrogen dioxide; O_3_: ozone; PM_10_ and PM_2.5_: particles with a 50% cutoff aerodynamic diameter of 10 µm and 2.5 µm, respectively

NO_2_ exposure tended to be associated with the presence of a limited ILD (adjusted OR 0.95, 95% CI 0.91–1.00; *p* value = 0.08). We found no association between the presence of extensive ILD and exposure to PMs. The association between extensive ILD at diagnosis and O_3_ exposure was confirmed in two-pollutant model (Table 3), whereas no association with NO_2_ exposure was found after adjustment for other pollutants exposure (OR after adjustment for O_3_ exposure: 1.09, 95% CI 0.99–1.24; *p* value = 0.21) (Additional file 1: Table S2).

**Table 3:** Association of pre-diagnosis exposure to ozone pollution with the severity of SSc-associated ILD at diagnosis (extensive ILD): two-pollutant model

	OR	*p* value
O_3_		
+ NO_2_	1.24 (1.08–1.51)	0.007
+ PM_10_	1.16 (1.06–1.30)	0.002
+ PM_2.5_	1.19 (1.06–1.34)	0.003

Logistic regression model adjusted for birth in Europe, tobacco smoking, anti-topoisomerase I antibodies positivity, usual interstitial pneumonia pattern, time between first non-Raynaud symptom and ILD diagnosis and year of ILD diagnosis

ILD: interstitial lung disease; NO_2_: nitrogen dioxide; O_3_: ozone; PM_10_ and PM_2.5_: particles with a 50% cutoff aerodynamic diameter of 10 µm and 2.5 µm, respectively

In the sensitivity analysis, exposure to O_3_ was associated with a TLC < 70% (adjusted OR: 1.07, 95% CI 1.01–1.15; *p* value = 0.03), and an extension on HRCT > 10% (adjusted OR: 1.07, 95% CI 1.01–1.14; *p* value = 0.03) (Table 4). Considering baseline functional and radiological data as continuous parameters in mixed linear models, exposure to O_3_ was also negatively associated with DLCO (Slope estimate: − 0.51 (Standard Error: 0.22), *t* value = 2.29, *p* value = 0.02) (Table 4).

**Table 4:** Association of air pollution (pre-diagnosis exposure) with the severity of systemic sclerosis-associated ILD at diagnosis: sensitivity analysis

	FVC < 70%^a^		TLC < 70%^a^		DLCO < 40%^a^		CPI > 40^a^		Extension > 10%^a^						
	OR	*p* value	OR	*p* value	OR	*p* value	OR	*p* value	OR						*p* value
NO_2_	0.99 (0.95–1.03)	0.72	0.98 (0.93–1.03)	0.33	0.97 (0.92–1.02)	0.18	1.00 (0.95–1.05)	0.91	0.96 (0.92–1.00)						0.06
O_3_	1.07 (0.98–1.10)	0.21	1.07 (1.01–1.15)	0.03	1.05 (0.98–1.11)	0.16	1.03 (0.97–1.11)	0.36	1.07 (1.01–1.14)						0.03
PM_10_	0.98 (0.83–1.16)	0.83	1.02 (0.83–1.27)	0.87	0.89 (0.73–1.08)	0.22	1.01 (0.83–1.25)	0.90	0.92 (0.78–1.07)						0.28
PM_2.5_	0.94 (0.72–1.23)	0.63	0.83 (0.59–1.18)	0.28	0.91 (0.67–1.25)	0.53	0.74 (0.51–1.03)	0.08	0.89 (0.67–1.16)						0.39

	FVC (% th)^b^			TLC (% th)^b^			DLCO (%th)^b^			CPI^b^			Extension^b^		
	Slope estimate (SE)	*t* value	*p* value	Slope estimate (SE)	*t* value	*p* value	Slope estimate (SE)	*t* value	*p* value	Slope estimate (SE)	*t* value	*p* value	Slope estimate (SE)	*t* value	*p* value
NO_2_	− 0.06 (0.20)	− 0.29	0.78	− 0.09 (0.19)	− 0.49	0.63	0.32 (0.17)	1.88	0.06	− 0.13 (0.15)	− 0.88	0.38	− 0.13 (0.11)	− 1.10	0.27
O_3_	− 0.02 (0.27)	− 0.06	0.96	− 0.10 (0.25)	− 0.39	0.70	− 0.51 (0.22)	− 2.29	0.02	0.31 (0.20)	1.56	0.12	0.22 (0.16)	1.39	0.17
PM_10_	0.30 (0.79)	0.38	0.71	− 0.07 (0.75)	− 0.09	0.93	1.29 (0.67)	1.93	0.06	− 0.54 (0.59)	− 0.92	0.36	− 0.15 (0.45)	− 0.34	0.73
PM_2.5_	0.43 (1.22)	0.36	0.72	0.21 (1.20)	0.17	0.86	2.07 (1.05)	1.97	0.05	− 1.87 (0.92)	− 2.03	0.04	0.33 (0.74)	0.45	0.65

CPI: composite physiological index; DLCO: diffusion capacity for carbon monoxide across the lung; FEV1: forced expiratory volume in 1 s; FVC: forced vital capacity; ILD: interstitial lung disease; NO_2_: nitrogen dioxide; O_3_: ozone; PM_10_ and PM_2.5_: particles with a 50% cutoff aerodynamic diameter of 10 µm and 2.5 µm, respectively; TLC: total lung capacity

^a^Logistic regression models

^b^Multiple linear models. Models adjusted for: Anti-topoisomerase I Abs positivity, time between first non-Raynaud symptom and ILD diagnosis, and year of ILD diagnosis for FVC and ILD extent on HRCT; socio-economic status of worker, anti-topoisomerase I Abs positivity, time between first non-Raynaud symptom and ILD diagnosis, and year of ILD diagnosis for TLC; time between first non-Raynaud symptom and ILD diagnosis, and year of ILD diagnosis for CPI and DLCO

### ILD evolution and air pollution exposure

Among 167 patients with at least one PFT during follow-up, median (IQR) FVC decline was − 33.4 (− 102; 19.9) mL/year, and median DLCO decline − 1 (− 3.7; 0.5) % of predicted/year. Among patients with PFT at 12 and 24 months, a FVC decline ≥ 10% was observed in 18/97 (18.6%) patients at 12 months and 17/89 (19.1%) patients at 24 months; a DLCO decline ≥ 15% was observed in 12/73 (16.4%) patients at 12 months and 18/65 (27.7%) patients at 24 months (Additional file 1: Table S3). Progression was observed in 27/105 (25.7%) patients at 12 months and 48/113 (42.5%) patients at 24 months (including 8 and 24 deaths respectively, no pulmonary transplantation). Radiological progression was noted in 54/141 patients (38.3%) at 24 months. After a median follow-up of 4.7 years (IQR 2.4–8.0 years), 24 patients (13.3%) had died and two had undergone lung transplantation.

**Fig. 3:**
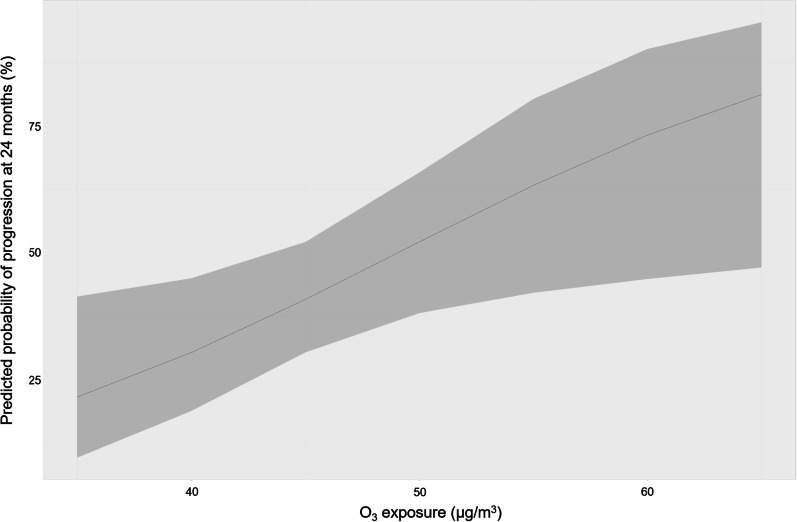
Predicted probability of progression at 24 months according to ozone exposure in final multivariate logistic regression model

In univariate analysis, O_3_ exposure during the year of ILD diagnosis was associated with progression at 24 months (OR: 1.08, 96% CI 1.01–1.15; *p* value = 0.03) (Additional file 1: Table S4). Parameters included in the final multivariate logistic regression model were those associated with progression at 24 months in multivariate analysis: age at ILD diagnosis (adjusted OR: 1.05, 95% CI 1.01–1.10; *p* value = 0.02), socio-professional status of worker (adjusted OR: 3.47, 95% CI 0.80–16.77; p value = 0.10), dcSSc (adjusted OR: 2.22, 95% CI 0.78–6.65; *p* value = 0.14), and anti-topoisomerase I antibodies positivity (adjusted OR: 4.97, 95% CI 1.52–18.88;* p* value = 0.01). In the final multivariate model, O_3_ exposure was significantly associated with progression at 24 months (adjusted OR: adjusted OR: 1.10, 95% CI 1.02–1.19; *p* value = 0.02) (Table 5 and Additional file 1: Table S4). Thus, the predicted probability of progression at 24 months for mean exposures of 35 µg/m^3^ and 65 µg/m^3^ were respectively 25 (95% CI 13–44) % and 76 (95% CI 41–93) % (Fig. 3).

**Table 5:** Association of air pollution (year of diagnosis exposure) with progression at 12 and 24 months

	12 months		24 months	
	OR	*p* value	OR	*p* value
NO_2_	0.96 (0.90–1.01)	0.14	0.96 (0.10–1.02)	0.19
O_3_	1.04 (0.96–1.12)	0.35	1.10 (1.02–1.19)	0.02
PM_10_	0.97 (0.84–1.10)	0.61	0.93 (0.83–1.04)	0.18
PM_2.5_	0.94 (0.77–1.13)	0.51	0.89 (0.75–1.04)	0.15

Logistic regression models adjusted for age at ILD diagnosis, socio-professional status of worker, diffuse cutaneous scleroderma, anti-topoisomerase I antibodies positivity

NO_2_, nitrogen dioxide; O_3_, ozone; PM_10_ and PM_2.5_, particles with a 50% cutoff aerodynamic diameter of 10 µm and 2.5 µm, respectively

**Fig. 4:**
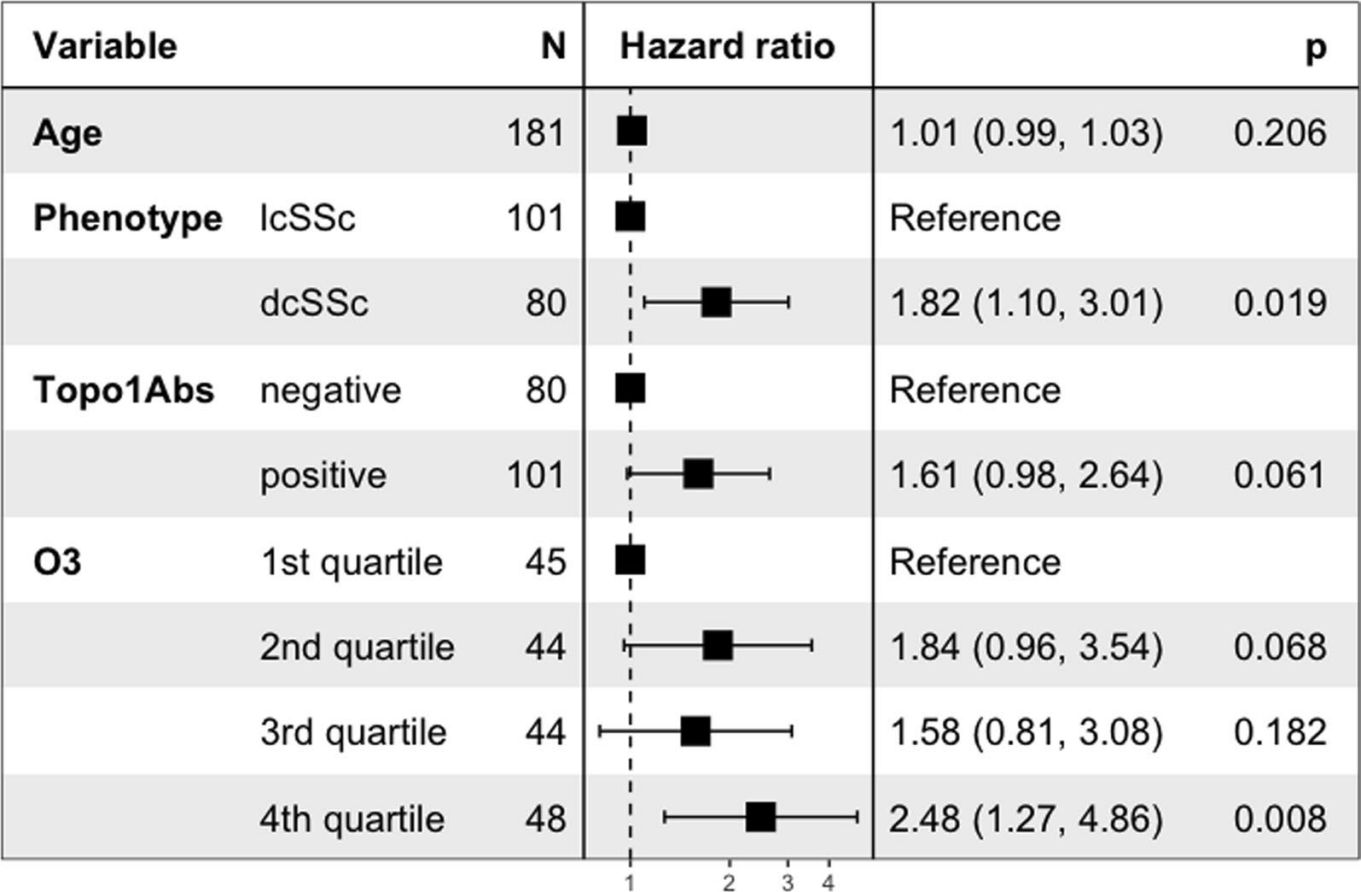
Forest plot showing the results of multivariate Cox proportional hazards model for ILD progression within 24 months following SSc-associated ILD diagnosis. dcSSc: diffuse cutaneous scleroderma, lcSSc: limited cutaneous scleroderma; O_3_: Ozone exposure on the year of ILD diagnosis; Topo1Abs: anti-topoisomerase I antibodies

No significant association was found between air pollution exposure on the year of ILD diagnosis and the occurrence of progression at 12 months. O_3_ exposure was significantly associated with the risk of progression within 24 months following ILD diagnosis in Cox proportional risk model: HR 1.04, 95% CI 1.00–1.08, *p* value = 0.03. Categorical analysis by quartiles of O_3_ exposure yielded a hazard ratio of 2.48 (95% CI 1.27–4.86, *p* value = 0.008) for patient in the fourth quartile compared to the first quartile (Fig. 4).

In the sensitivity analysis, O_3_ exposure was associated with a FVC decline ≥ 5% (adjusted OR: 1.15, 95% CI 1.03–1.29; *p* value = 0.02) at 24 months (Additional file 1: Table S5). No association was found between air pollution exposure and a decline of DLCO ≥ 15%, ≥ 10%, or ≥ 7.5% at 24 months (Additional file 1: Table S5) or radiological progression (Additional file 1: Table S6).

In linear mixed models, no significant association was found between air pollution exposure and change in FVC or DLCO over time. No association was found between air pollution exposure at time of ILD diagnosis and transplant-free survival (Additional file 1: Table S7).

## Discussion

We investigated the association between the severity of SSc-associated ILD and chronic exposure to PM_2.5_, PM_10_, NO_2_ and O_3_ in a cohort of SSc patients seen at two hospitals in the Paris area. We observed an association between long-term exposure to O_3_ and ILD severity at diagnosis, evaluated with the Goh staging algorithm, and according to extension on HRCT, TLC and DLCO. This association was independent of the principal factors associated with the severity of SSc-associated ILD and was confirmed in two-pollutant models. We also found an association between O_3_ exposure and progression at 24 months. We found no association between exposure to other pollutants and severity at diagnosis and progression.

Ozone is a secondary pollutant generated principally by the photochemical reaction of nitric oxides and oxygen molecules in the atmosphere. It has detrimental effects at concentrations only three to four times higher than natural background levels [32]. Episodes of high O_3_ concentration occur in urbanized areas during periods of sunny anticyclonic weather in the summer months. O_3_ is a highly reactive gas, and a powerful oxidant. Epidemiological studies have shown chronic O_3_ exposure to be associated with the risk of death from respiratory causes [33], and the incidence and mortality of acute respiratory distress syndrome [34, 35]. Animal models and lung autopsy study have revealed the presence of chronic epithelial changes, including fibrosis, in subjects chronically exposed to high O_3_ concentrations [36, 37]. In its Integrated Science Assessment for Ozone, the United States Environmental Protection Agency estimated that there is a “causal relationship” and a “likely causal relationship” between short-term and long-term O_3_ exposure, respectively, and respiratory effects [38]. In IPF, the onset of an acute exacerbation has been shown to be associated with an increase in O_3_ exposure within the preceding 6 weeks; however, no association between long-term O_3_ exposure and IPF severity has ever been reported [13]. Nevertheless, long-term exposure to O_3_ has been shown to be positively associated with serum IL-4 levels in IPF patients, and tends to be associated with osteopontin levels, two mediators implicated in fibrosis [39].

The role of air pollution in autoimmune diseases has been studied essentially in rheumatoid arthritis (RA). Particulate matter, such as diesel emission particles, is thought to induce the citrullination of lung proteins and the development of inducible bronchus-associated lymphoid tissue (iBALT), leading to the production of pathogenic anti-citrullinated protein antibodies (ACPA) [40, 41]. iBALT hyperplasia and the activation of T-cells contained in pulmonary lymph nodes have been observed in animal models exposed to O_3_ [42, 43]. In SSc patients, exposure to O_3_ may trigger the development of iBALT-inducing pathogenic autoantibodies. Lung oxidant/antioxidant equilibrium is disturbed at high levels of O_3_ exposure, or in situations in which the lung lining fluid antioxidant power is compromised. The reaction of O_3_ with substrates present in the lung lining fluid compartment then generates secondary oxidation products and inflammation [32, 44]. Reactive oxygen species (ROS) have profibrogenic effects on fibroblasts and induce the release of profibrotic mediators, such as transforming growth factor-β 1 (TGF β 1) [45]. High levels of ROS, produced by the NADPH oxidase system, have been implicated in the pathophysiology of SSc [46–48]. Scleroderma fibroblasts cannot respond to oxidative stress and they mount an inadequate antioxidant response [46].

Borghini et al. reported that exposure to benzene was inversely correlated with DLCO and positively correlated with Rodnan skin score in SSc patients, whereas they found no association with PM_10_ exposure [21]. Benzene is mostly emitted during wood heating in human homes and in the transport sector and contribute to the formation of O_3_ through reactions involving nitrogen oxides (NOx) and solar radiation. Recently, Goobie et al. reported the association of PM_2.5_ exposure with lung function at baseline and mortality among patients with fibrotic ILDs [49]. Homogeneity of particulate matter exposure among our patients could have limited the evaluation of their impact on ILD severity and progression. To our knowledge, ours is the first study to evaluate the effect of O_3_ exposure in SSc patients.

Our work has several limitations. First, due to the rarity of SSc and the two-center design of the study, the number of patients included was small for the purpose to detect correlations. Most of the patients were living in the same region, limiting the variability of exposure. However, the use of the CHIMERE model increased the accuracy of exposure estimates, making it possible to detect smaller differences in exposure than would have been possible with the use of concentration data from air quality stations. A limitation inherent to the study design is the estimation of personal exposure at residential addresses, while exposures take place in multiple locations. Assuming that the error in the estimates is random, it would likely bias any association to zero, suggesting that the true magnitude of the effect may be greater than measured [50]. Mean annual O_3_ exposure were considered in our analysis, while the maximum O_3_ concentrations are reached during the daytime period in the summer months. Thus, the association of high O_3_ levels with respiratory effects may have been underestimated. Trends in the exposure to air pollutants over time may be a source of confounding. However, concentrations of particulate matters and NO_2_ have fallen over the years, whereas concentrations of O_3_ have increased in our study population, whereas the trend over the years regarding ILD in SSc patients may be assumed toward an earlier diagnosis through HRCT and a better outcome. Moreover, our results were consistent after adjustment for the year of ILD diagnosis. The study was retrospective. As a result, evaluation was not standardized and many PFT data were missing at 12 and 24 months, limiting evaluations of the effects of pollution on progression in our study. Inclusion period (2006–2019) limited follow-up time and the interpretation of the results of survival data. Most of the patients included were followed in the French referral center for SSc (Internal medicine department, Cochin Hospital) or the competence center for ILDs (Respiratory medicine department, Avicenne Hospital), and may therefore not represent a general SSc population. Last but not least, our study’s primary objective was to determine the association between SSc-associated ILD severity at diagnosis but did not consider the incidence of ILD in SSc patients. We did not study the effect of exposure to pollution on extrapulmonary SSc manifestations. Therefore, the role of air pollution exposure on ILD occurrence and other organ involvements in SSc patients remains to be determined.

Despite the retrospective nature of this study, HRCT characteristics at ILD diagnosis, reviewed by expert radiologists, were available for all the patients included, together with PFT parameters, allowing an accurate evaluation of ILD at diagnosis. Our study showed consistently significant associations with O_3_, however, supportive evidence from future studies in various geographic areas and animal models describing pathophysiological pathways implicated will be necessary to strengthen the arguments for causality.

In conclusion, this study is the first to assess the impact of air pollution on SSc-associated ILD. It reveals an association between O_3_ exposure and ILD severity at diagnosis and progression at 24 months, that is independent of the principal factors associated with disease severity and progression. The identification of this preventable risk factor could lead to avoidance measures, particularly during periods of high O_3_ levels in warm weather. A prospective larger-scale multicenter study with a standardized evaluation of progression and prolonged follow-up is required, to confirm our results and to assess the effect of air pollution exposure on SSc-associated ILD incidence and outcome.

## Supplementary Information

**Additional file 1: Table S1.** Factors associated with the severity at diagnosis of systemic sclerosis associated interstitial lung disease. **Table S2.** Association of air pollution with the severity of SSc-associated ILD at diagnosis: two pollutant-models. **Table S3.** Functional changes during follow-up. **Table S4.** Factors associated with the evolution of systemic sclerosis associated interstitial lung disease. **Table S5.** Association of air pollution with categorial changes in pulmonary function test results at 24 months. **Table S6.** Association of air pollution with radiological progression at 24 months. **Figure S1.** Flow chart.

## Data Availability

The datasets used and/or analyzed during the current study are available from the corresponding authors (Y.U.) on reasonable request.

## Abbreviations

dcSSc: Diffuse cutaneous systemic sclerosis
DLCO: Diffusing capacity of the lung for carbon monoxide
FEV1: Forced expiratory volume in 1 s
FVC: Forced vital capacity
HRCT: High-resolution computed tomography
ILD: Interstitial lung disease
IPF: Idiopathic pulmonary fibrosis
lcSSc: Limited cutaneous systemic sclerosis
NO_2_: Nitrogen dioxide
NSIP: Non-specific interstitial pneumonia
O_3_: Ozone
OP: Organizing pneumonia
PFT: Pulmonary function test
PM_2.5_: Particulate matter of up to 2.5 µm in diameter
PM_10_: Particulate matter of up to 10 µm in diameter
ROS: Reactive oxygen species
SSc: Systemic sclerosis
TLC: Total lung capacity
UIP: Usual interstitial pneumonia
