# Supplementary material for: The association between air pollution and the severity at diagnosis and progression of systemic sclerosis-associated interstitial lung disease: results from the retrospective ScleroPol study
Source: Respir Res. 2023 Jun 8;24:151. doi: 10.1186/s12931-023-02463-w (PMC10249172; doi:10.1186/s12931-023-02463-w)
Supplement: Supplementary file 1 — Additional file 1: Table S1. Factors associated with the severity at diagnosis of systemic sclerosis associated interstitial lung disease. Table S2. Association of air pollution with the severity of SSc-associated ILD at diagnosis: two pollutant-models. Table S3. Functional changes during follow-up. Table S4. Factors associated with the evolution of systemic sclerosis associated interstitial lung disease. Table S5. Association of air pollution with categorial changes in pulmonary function test results at 24 months. Table S6. Association of air pollution with radiological progression at 24 months. Figure S1. Flow chart. [file 12931_2023_2463_MOESM1_ESM.docx]

**ADDITIONAL FILE 1**

**Table S1. Factors associated with the severity at diagnosis of systemic sclerosis associated interstitial lung disease (SSc-ILD)**

|  | **Extensive ILD*** (Goh staging) | | | | **Baseline CPI**° | | | | **Baseline DLCO°** | | | |
| --- | --- | --- | --- | --- | --- | --- | --- | --- | --- | --- | --- | --- |
|  | *Univariate analysis* | | *Final multivariate model* | | *Univariate analysis* | | *Final multivariate model* | | *Univariate analysis* | | *Final multivariate model* | |
|  | OR (95%CI) | *p* value | OR (95%CI) | *p* value | Slope Estimate (SE) | *t* value | Slope Estimate (SE) | *t* value | Slope Estimate (SE) | *t* value | Slope Estimate (SE) | *t* value |
| Female sex | 0.71 (0.33-1.56) | 0.38 | **-** | **-** | **-4.75 (3.27)** | **-1.45** | - | - | **5.39 (3.71)** | **1.45** | - | - |
| Birth in Europe | **0.34 (0.17-0.65)** | **0.001** | 0.28 (0.11-0.67) | 0.005 | **-3.66 (2.62)** | **-1.40** | - | - | 0.87 (3.01) | 0.29 | - | - |
| Age at ILD diagnosis | 0.99 (0.97-1.01) | 0.45 | - | - | -0.03 (0.09) | -0.37 | - | - | -0.05 (0.10) | -0.51 | - | - |
| Tobacco smoking | **0.56 (0.26-1.13)** | **0.12** | 0.31 (0.10-0.89) | 0.04 | **4.70 (2.79)** | **1.68** | - | - | **-7.46 (3.08)** | **-2.42** | - | - |
| Working class | 1.51 (0.59-3.67) | 0.37 | - | - | **5.13 (3.62)** | **1.42** | - | - | **-6.66 (4.14)** | **-1.61** | - | - |
| Diffuse cutaneous SSc | 1.32 (0.69-2.52) | 0.40 | - | - | **5.51 (2.59)** | **2.13** | - | - | **-4.37 (2.95)** | **-1.48** | - | - |
| Anti-topo I Ab | **2.20 (1.13-4.45)** | **0.02** | 4.95 (1.87-14.86) | 0.002 | 2.22 (2.62) | 0.85 | - | - | 0.01 (2.95) | 0.004 | - | - |
| UIP pattern | **2.19 (0.67-6.92)** | **0.18** | 3.73 (0.68-20.20) | 0.12 | **6.47 (4.43)** | **1.46** | - | - | -4.31 (5.41) | -0.80 | - | - |
| Time between first non-Raynaud symptom and ILD diagnosis | **0.93 (0.85-1.00)** | **0.08** | 0.93 (0.83-1.01) | 0.14 | **-0.74 (0.21)** | **-3.62** | -0.85 (0.20) | -4.33 | **0.78 (0.23)** | **3.40** | 0.86 (0.22) | 3.87 |
| Year of ILD diagnosis | 1.05 (0.96-1.15) | 0.30 | 1.08 (0.97-1.22) | 0.19 | **0.82 (0.35)** | **2.33** | 1.13 (0.33) | 3.39 | **-0.73 (0.40)** | **-1.84** | -1.04 (0.39) | -2.63 |
| O_3_ exposure | **1.05 (1.00-1.11)** | **0.06** | 1.12 (1.05-1.21) | 0.002 | **0.40 (0.21)** | **1.87** | 0.31 (0.20) | 1.56 | -0.58 (0.23) | 0.01 | -0.51 (0.22) | -2.29 |

|  | **Baseline FVC (%)**° | | | | **Baseline TLC (%)**° | | | | **Baseline ILD extent on HRCT**° | | | |
| --- | --- | --- | --- | --- | --- | --- | --- | --- | --- | --- | --- | --- |
|  | *Univariate analysis* | | *Final multivariate model* | | *Univariate analysis* | | *Final multivariate model* | | *Univariate analysis* | | *Final multivariate model* | |
|  | Slope Estimate (SE) | *t* value | Slope Estimate (SE) | *t* value | Slope Estimate (SE) | *t* value | Slope Estimate (SE) | *t* value | Slope Estimate (SE) | *t* value | Slope Estimate (SE) | *t* value |
| Female sex | **6.37 (4.09)** | **1.56** | **-** | **-** | **5.49 (3.69)** | **1.49** | **-** | **-** | 0.80 (2.28) | 0.35 | - | - |
| Birth in Europe | **11.38 (3.22)** | **3.54** | **-** | **-** | **7.06 (3.07)** | **2.30** | **-** | **-** | **-2.59 (1.86)** | **-1.39** | - | - |
| Age at ILD diagnosis | **0.33 (0.11)** | **3.04** | **-** | **-** | 0.12 (0.11) | 1.17 | **-** | **-** | 0.02 (0.06) | 0.35 | - | - |
| Tobacco smoking | 3.51 (3.56) | 0.99 | **-** | **-** | 0.32 (3.24) | 0.10 | - | - | - 1.07 (2.01) | -0.53 | - | - |
| Working class | -2.94 (4.86) | -0.60 | **-** | **-** | **-9.44 (4.16)** | **-2.27** | -11.2 (4.60) | -2.44 | 0.45 (2.63) | 0.17 | - | - |
| Diffuse cutaneous SSc | **-6.34 (3.26)** | **-1.94** | - | - | **-5.97 (3.05)** | **-1.96** | - | - | **2.74 (1.84)** | **1.49** | - | - |
| Positive anti-topo I Ab | **-9.11 (3.26)** | **-2.79** | -10.30 (3.38) | -3.05 | **-5.25 (3.12)** | **-1.69** | -7.34 (3.32) | -2.21 | **5.87 (1.83)** | **3.22** | 6.49 (1.95) | 3.34 |
| UIP pattern | -7.50 (6.51) | -1.15 | - | - | **-9.71 (6.13)** | **-1.58** | - | - | 3.20 (3.55) | 0.90 | - | - |
| Time between first non-Raynaud symptom and ILD diagnosis | **1.10 (0.27)** | **4.00** | 1.06 (0.27) | 3.92 | **0.84 (0.27)** | **3.08** | 0.75 (0.27) | 2.77 | **-0.46 (0.16)** | **-2.90** | -0.42 (0.16) | -2.71 |
| Year of ILD diagnosis | **-0.54 (0.44)** | **-1.23** | -0.80 (0.44) | -1.79 | -0.45 (0.41) | -1.10 | -0.55 (0.41) | -1.34 | 0.08 (0.25) | 0.34 | 0.21 (0.26) | 0.82 |
| O_3_ exposure | -0.06 (0.28) | -0.23 | -0.02 (0.27) | -0.06 | -0.14 (0.25) | -0.57 | -0.10 (0.25) | -0.39 | **0.22 (0.15)** | **1.44** | 0.22 (0.16) | 1.39 |

Univariate analysis and final multivariate models including ozone exposure (ozone exposure: mean exposure on 5 years before ILD diagnosis). *Logistic regression models. °Mixed linear models. Anti-topo I Ab: anti-topoisomerase I antibodies; DLCO: diffusion capacity for carbon monoxide across the lung; FVC: forced vital capacity; HRCT: high resolution computed tomography; ILD: interstitial lung disease; SSc: systemic sclerosis; UIP: usual interstitial pneumoniae.

|  | OR | *p* value |
| --- | --- | --- |
| **NO_2_**  + O_3_  + PM_10_  + PM_2.5_ | 1.09(0.99-1.24)  0.92 (0.83-1.01)  0.98 (0.90-1.07) | 0.123  0.067  0.704 |
| **O_3_**  + NO_2_  + PM_10_  + PM_2.5_ | **1.24 (1.08-1.51)**  **1.16 (1.06-1.30)**  **1.19 (1.06-1.34)** | **0.007**  **0.002**  **0.003** |
| **PM_10_**  + NO_2_  + O_3_ | 1.21 (0.85-1.76)  1.18 (0.92-1.59) | 0.305  0.227 |
| **PM_2.5_**  + NO_2_  + O_3_ | 0.94 (0.57-1.54)  1.48 (0.92-2.54) | 0.800  0.127 |

**Table S2. Association of air pollution (pre-diagnosis exposure) with the severity of SSc-associated ILD at diagnosis (extensive ILD): two pollutant-models**

Logistic regression models adjusted for birth in Europe, tobacco smoking, anti-topoisomerase I antibodies positivity, usual interstitial pneumonia pattern, time between first non-Raynaud symptom and ILD diagnosis, **and year of ILD diagnosis**.

ILD: interstitial lung disease; NO_2_: nitrogen dioxide; O_3_: ozone; PM_10_ and PM_2.5_: particles with a 50% cutoff aerodynamic diameter of 10 µm and 2.5 µm, respectively.

**Table S3. Functional changes during follow-up**

|  | **12 months** | **24 months** |
| --- | --- | --- |
| **FVC** | *N*=97 | *N*=89 |
| Improvement (+ΔFVC > 3 %), *n* (%) | 29 (29.9) | 36 (40.4) |
| Stable, *n* (%) | 35 (36.1) | 25 (28.1) |
| Moderate decline (-ΔFVC between 5% and 10%), *n* (%) | 15 (15.5) | 11 (12.4) |
| Significant decline (-ΔFVC ≥ 10 %), *n* (%) | 18 (18.6) | 17 (19.1) |
| **DLCO** | *N*=73 | *N*=65 |
| Improvement (+ΔDLCO > 3 %), *n* (%) | 27 (37.0) | 20 (30.8) |
| Stable, *n* (%) | 21 (28.8) | 20 (30.8) |
| Moderate decline (-ΔDLCO between 7.5% and 15%), *n* (%) | 13 (17.8) | 7 (10.7) |
| Significant decline (-ΔDLCO ≥ 15 %), *n* (%) | 12 (16.4) | 18 (27.7) |

DLCO: diffusion capacity for carbon monoxide across the lung; ΔDLCO: relative change in DLCO (expressed as a percentage of the theoretical value); FVC: forced vital capacity; ΔFVC: relative change in FVC (expressed in liters).

**Table S4. Factors associated with the evolution of systemic sclerosis associated interstitial lung disease (SSc-ILD)**

|  | **Progression at 12months*** | | | | **Progression at 24months*** | | | | **Time to progression^#^** | | | |
| --- | --- | --- | --- | --- | --- | --- | --- | --- | --- | --- | --- | --- |
|  | *Univariate analysis* | | *Final multivariate model* | | *Univariate analysis* | | *Final multivariate model* | | *Univariate analysis* | | *Final multivariate model* | |
|  | OR (95%CI) | *p* value | OR (95%CI) | *p* value | OR (95%CI) | *p* value | OR (95%CI) | *p* value | HR (95%CI) | *p* value | HR (95%CI) | *p* value |
| Female sex | 1.23 (0.43-4.08) | 0.72 | - | - | 0.61 (0.22-1.65) | 0.33 | - | - | 1.30 (0.69-2.50) | 0.42 | - | - |
| Birth in Europe | 1.31 (0.54-3.32) | 0.55 | - | - | 1.61 (0.75-3.54) | 0.23 | - | - | **1.70 (1.00-2.70)** | **0.03** | - | - |
| Age at ILD diagnosis | **1.03 (1.00-1.06)** | **0.06** | 1.05 (1.01-1.09) | 0.02 | **1.02 (1.00-1.05)** | **0.12** | 1.07 (1.03-1.12) | 0.002 | 1.00 (0.98-1.00) | 0.56 | 1.01 (0.99-1.03) | 0.27 |
| Tobacco smoking | 0.84 (0.31-2.14) | 0.72 | - | - | 1.47 (0.65-3.34) | 0.36 | - | - | 1.20 (0.73-1.90) | 0.51 | - | - |
| Working class | 0.83 (0.21-2.63) | 0.76 | 1.10 (0.26-4.15) | 0.90 | **2.83 (0.98-8.87)** | **0.06** | 11.05 (2.46-61.32) | 0.003 | 1.20 (0.66-2.10) | 0.57 | - | - |
| Diffuse cutaneous SSc | 1.61 (0.67-4.00) | 0.29 | 2.16 (0.80-6.13) | 0.14 | **2.06 (0.97-4.44)** | **0.06** | 3.86 (1.45-11.24) | 0.009 | **1.70 (1.10-2.60)** | **0.03** | 1.67 (1.03-2.72) | 0.04 |
| Positive anti-topo I Ab | 1.42 (0.57-3.70) | 0.4 | 2.28 (0.74-7.62) | 0.16 | **1.66 (0.77-3.63)** | **0.19** | 8.37 (2.46-34.90) | 0.002 | **1.50 (0.95-2.40)** | **0.08** | 1.61 (0.98-2.63) | 0.06 |
| UIP pattern | 0.00 (NA-1.10^44^) | 0.991 | - | - | **2.90 (0.72-14.37)** | **0.15** | - | - | **0.47 (0.17-1.30)** | **0.15** | - | - |
| Baseline FVC (%th) | 1.01 (0.99-1.03) | 0.20 | - | - | 1.01 (0.99-1.02) | 0.42 | - | - | 1.00 (0.99-1.00) | 0.95 | - | - |
| Baseline DLCO (%th) | 0.99 (0.97-1.02) | 0.57 | - | - | **0.98 (0.95-1.00)** | **0.05** | - | - | **0.99 (0.97-1.00)** | **0.04** | - | - |
| ILD extension on HRCT | 1.01 (0.98-1.05) | 0.38 | - | - | 1.02 (0.99-1.05) | 0.30 | - | - | **1.00 (1.00-1.00)** | **0.10** | - | - |
| Time between first non-Raynaud symptom and ILD diagnosis | 1.00 (0.92-1.08) | 0.94 | - | - | **0.95 (0.88-1.02)** | **0.18** | - | - | **0.96 (0.92-1.00)** | **0.08** | - | - |
| Immunosuppressor initiation | 1.02 (0.42-2.49) | 0.97 | - | - | 1.19 (0.56-2.53) | 0.65 | - | - | **1.50 (0.96-2.40)** | **0.07** | - | - |
| Year of ILD diagnosis | 1.01 (0.89-1.15) | 0.88 | - | - | 1.07 (0.96-1.19) | 0.25 | - | - | **1.00 (0.94-1.10)** | **0.96** | - | - |
| O_3_ exposure | 1.04 (0.96-1.11) | 0.35 | 1.05 (0.97-1.14) | 0.25 | **1.08 (1.01-1.15)** | **0.03** | 1.10 (1.02-1.19) | 0.02 | **1.00 (1.00-1.10)** | **0.04** | 1.04 (1.00-1.08) | 0.03 |

|  | **Change in FVC (mL)°** | | | | **Change in DLCO (%)°** | | | | **Transplant free survival^#^** | | | |
| --- | --- | --- | --- | --- | --- | --- | --- | --- | --- | --- | --- | --- |
|  | *Univariate analysis* | | *Final multivariate model* | | *Univariate analysis* | | *Final multivariate model* | | *Univariate analysis* | | *Final multivariate model* | |
|  | Slope Estimate (SE) | *t* value | Slope Estimate (SE) | *t* value | Slope Estimate (SE) | *t* value | Slope Estimate (SE) | *t* value | HR (95%CI) | *p* value | HR (95%CI) | *p* value |
| Female sex | -75.98 (66.83) | -1.14 | - | - | -1.93 (2.58) | -0.75 | - | - | **0.36 (0.15-0.86)** | **0.02** | 0.37 (0.12-1.17) | 0.09 |
| Birth in Europe | **-124.78 (52.77)** | **-2.37** | - | - | -0.11 (2.08) | -0.05 | - | - | 0.64 (0.28-1.50) | 0.29 | - | - |
| Age at ILD diagnosis | -0.31 (1.80) | -0.17 | - | - | 0.02 (0.07) | 0.27 | - | - | **1.00 (1.00-1.10)** | **0.05** | 1.05 (1.00-1.10) | 0.04 |
| Working class | **-110.30 (72.49)** | **-1.52** | -145.57 (65.29) | -2.23 | -0.99 (2.70) | -0.37 | - | - | **3.70 (1.50-9.2)** | **0.005** | - | - |
| Tobacco smoking | -10.67 (56.85) | -0.19 | - | - | 1.73 (2.11) | 0.82 | - | - | 1.60 (0.67-3.8) | 0.29 | - | - |
| Diffuse cutaneous SSc | 18.29 (52.96) | 0.35 | - | - | -1.64 (2.02) | -0.81 | - | - | 0.79 (0.33-1.90) | 0.60 | - | - |
| Positive anti-topo I Ab | -12.63 (53.95) | -0.23 | - | - | -1.17 (2.05) | -0.57 | - | - | **0.31 (0.13-0.78)** | **0.01** | 0.28 (0.09-0.84) | 0.02 |
| UIP pattern | 42.15 (113.07) | 0.37 | - | - | -0.36 (3.98) | -0.09 | - | - | **4.80 (1.60-15.0)** | **0.01** | - | - |
| Baseline FVC (%th) | **-6.70 (1.08)** | **-6.16** | -6.86 (1.08) | -6.36 | -0.05 (0.05) | -1.07 | - | - | 0.99 (0.97-1.0) | 0.41 | - | - |
| Baseline DLCO (%th) | -1.49 (1.49) | -1.00 | - | - | **-0.22 (0.05)** | **-4.28** | -0.21 (0.05) | -4.10 | **0.93 (0.89-0.96)** | **<0.01** | 0.94 (0.91-0.98) | 0.01 |
| ILD extension on HRCT | 0.83 (2.06) | 0.41 | - | - | 0.05 (0.08) | 0.67 | - | - | **1.00 (1.00-1.10)** | **0.02** | 1.05 (1.01-1.10) | 0.02 |
| Time between first non-Raynaud symptom and ILD diagnosis | -6.13 (5.02) | -1.22 | - | - | -0.07 (0.18) | -0.38 | - | - | 0.95 (0.84-1.10) | 0.35 | - | - |
| Immunosuppressor initiation | 28.15 (52.83) | 0.53 | - | - | -0.49 (2.02) | -0.24 | - | - | 0.92 (0.39-2.16) | 0.85 | - | - |
| Year of ILD diagnosis | -3.46 (7.46) | 0.46 | - | - | 0.14 (0.29) | 0.50 | - | - | **1.10 (0.97-1.30)** | **0.13** | - | - |
| O_3_ exposure | 0.09 (4.10) | 0.02 | - 3.72 (3.62) | -1.03 | 0.11 (0.15) | 0.74 | -0.01 (0.14) | -0.05 | 1.00 (0.94-1.07) | 0.93 | 0.99 (0.93-1.06) | 0.82 |

Univariate analysis and final multivariate models including ozone exposure (ozone exposure: mean exposure on the year of ILD diagnosis).

*Logistic regression models. °Mixed linear models. ^#^Cox proportional hazards models.

Anti-topo I Ab: anti-topoisomerase I antibodies; DLCO: diffusion capacity for carbon monoxide across the lung; FVC: forced vital capacity; HRCT: high resolution computed tomography; ILD: interstitial lung disease; SSc: systemic sclerosis; UIP: usual interstitial pneumoniae.

**Table S****5. Association of air pollution (year of ILD diagnosis exposure) with categorial changes in pulmonary function test results at 24 months**

|  | **FVC decline ≥ 10%** | | **FVC decline** **≥ 5%** | | **DLCO decline ≥ 15%** | | **DLCO decline ≥ 10%** | | **DLCO decline ≥ 7.5%** | |
| --- | --- | --- | --- | --- | --- | --- | --- | --- | --- | --- |
|  | OR | *p* value | OR | *p* value | OR | *p* value | OR | *p* value | OR | *p* value |
| **NO_2_** | 0.96 (0.87-1.04) | 0.33 | 0.92 (0.85-1.00) | 0.04 | 0.96 (0.88-1.04) | 0.31 | 0.99 (0.92-1.05) | 0.66 | 1.00 (0.93-1.07) | 0.94 |
| **O_3_** | 1.13 (0.99-1.30) | 0.07 | **1.15 (1.03-1.29)** | **0.02** | 1.07 (0.96-1.19) | 0.24 | 1.02 (0.94-1.11) | 0.67 | 1.00 (0.91-1.10) | 0.99 |
| **PM_10_** | 0.95 (0.79-1.13) | 0.54 | 0.91 (0.77-1.05) | 0.21 | 0.90 (0.73-1.09) | 0.29 | 0.93 (0.80-1.08) | 0.34 | 0.94 (0.80-1.10) | 0.45 |
| **PM_2.5_** | 1.01 (0.77-1.30) | 0.97 | 0.91 (0.73-1.13) | 0.41 | 0.99 (0.77-1.27) | 0.94 | 0.92 (0.75-1.12) | 0.33 | 0.94 (0.75-1.17) | 0.59 |

Logistic regression models adjusted for age at ILD diagnosis, socio-professional status of worker, diffuse cutaneous scleroderma, anti-topoisomerase I antibodies positivity.

ILD: interstitial lung disease; NO_2_, nitrogen dioxide; O_3_, ozone; PM_10_ and PM_2.5_, particles with a 50% cutoff aerodynamic diameter of 10 µm and 2.5 µm, respectively.

**Table S6. Association of air pollution (year of ILD diagnosis exposure) with radiological progression at 24 months**

|  | OR | *p* value |
| --- | --- | --- |
| **NO_2_** | 1.00 (0.95-1.06) | 0.91 |
| **O_3_** | 1.00 (0.94-1.06) | 0.99 |
| **PM_10_** | 1.02 (0.90-1.15) | 0.77 |
| **PM_2.5_** | 1.00 (0.84-1.19) | 0.99 |

Logistic regression models adjusted for diffuse cutaneous scleroderma, anti-topoisomerase I antibodies positivity, baseline diffusion capacity for carbon monoxide across the lung (% predicted), baseline ILD extension on HRCT, time between first non-Raynaud symptom and ILD diagnosis.

ILD: interstitial lung disease; NO_2_, nitrogen dioxide; O_3_, ozone; PM_10_ and PM_2.5_, particles with a 50% cutoff aerodynamic diameter of 10 µm and 2.5 µm, respectively.

**Table S7. Association of air pollution (year of ILD diagnosis exposure) with transplant-free survival**

|  | HR (95% CI) | *p* value |
| --- | --- | --- |
| **NO_2_** | 1.02 (0.96-1.09) | 0.44 |
| **O_3_** | 0.99 (0.93-1.05) | 0.82 |
| **PM_10_** | 1.00 (0.87-1.14) | 0.99 |
| **PM_2.5_** | 1.00 (0.81-1.23) | 0.96 |

Cox proportional hazards models adjusted for sex, age at systemic sclerosis-ILD diagnosis, anti-topoisomerase I antibodies positivity, baseline diffusion capacity for carbon monoxide across the lung (% predicted), ILD extension on high resolution lung computed tomography.

ILD: interstitial lung disease; NO_2_, nitrogen dioxide; O_3_, ozone; PM_10_ and PM_2.5_, particles with a 50% cutoff aerodynamic diameter of 10 µm and 2.5 µm, respectively

Cases of SSc-ILD screened

*N*= 269

Cases excluded

*N*= 88

Age at diagnosis < 18 years; *n*= 1

ILD diagnosis before 2006; *n*= 39

Absence of PFT within 3 months of diagnosis; *n*= 36

Residence in overseas territories; *n*= 12

**Cases of SSc-ILD included**

*N*= 181

Lung function evolution

*N*= 167 with at least one follow-up PFT

**Figure S1. Flow chart**

ILD: interstitial lung disease; PFT: pulmonary function test; SSc: systemic sclerosis
